# Dysfunctional purinergic signaling correlates with disease severity in COVID-19 patients

**DOI:** 10.3389/fimmu.2022.1012027

**Published:** 2022-09-30

**Authors:** Anna Julia Pietrobon, Roberta Andrejew, Ricardo Wesley Alberca Custódio, Luana de Mendonça Oliveira, Juliete Nathali Scholl, Franciane Mouradian Emidio Teixeira, Cyro Alves de Brito, Talita Glaser, Julia Kazmierski, Christine Goffinet, Anna Claudia Turdo, Tatiana Yendo, Valeria Aoki, Fabricio Figueiró, Ana Maria Battastini, Henning Ulrich, Gill Benard, Alberto Jose da Silva Duarte, Maria Notomi Sato

**Affiliations:** ^1^ Laboratory of Dermatology and Immunodeficiencies, LIM-56, Department of Dermatology, Tropical Medicine Institute of São Paulo, University of São Paulo Medical School, São Paulo, Brazil; ^2^ Department of Immunology, Institute of Biomedical Sciences, University of São Paulo, São Paulo, Brazil; ^3^ Department of Biochemistry, Institute of Chemistry, University of São Paulo, São Paulo, Brazil; ^4^ Department of Biochemistry, Federal University of Rio Grande do Sul, Porto Alegre, Brazil; ^5^ Technical Division of Medical Biology, Immunology Center, Adolfo Lutz Institute, São Paulo, Brazil; ^6^ Institute of Virology, Charité - Universitätsmedizin Berlin, Berlin, Germany; ^7^ Department and Division of Infectious and Parasitic Diseases, Berlin Institute of Health, Berlin, Germany; ^8^ Department and Division of Infectious and Parasitic Diseases, Hospital das Clinicas, University of São Paulo Medical School, São Paulo, Brazil

**Keywords:** adenosine, ATP, CD39, CD73, COVID-19, SARS-CoV-2, purinergic signaling

## Abstract

Ectonucleotidases modulate inflammatory responses by balancing extracellular ATP and adenosine (ADO) and might be involved in COVID-19 immunopathogenesis. Here, we explored the contribution of extracellular nucleotide metabolism to COVID-19 severity in mild and severe cases of the disease. We verified that the gene expression of ectonucleotidases is reduced in the whole blood of patients with COVID-19 and is negatively correlated to levels of CRP, an inflammatory marker of disease severity. In line with these findings, COVID-19 patients present higher ATP levels in plasma and reduced levels of ADO when compared to healthy controls. Cell type-specific analysis revealed higher frequencies of CD39+ T cells in severely ill patients, while CD4+ and CD8+ expressing CD73 are reduced in this same group. The frequency of B cells CD39+CD73+ is also decreased during acute COVID-19. Interestingly, B cells from COVID-19 patients showed a reduced capacity to hydrolyze ATP into ADP and ADO. Furthermore, impaired expression of ADO receptors and a compromised activation of its signaling pathway is observed in COVID-19 patients. The presence of ADO in vitro, however, suppressed inflammatory responses triggered in patients’ cells. In summary, our findings support the idea that alterations in the metabolism of extracellular purines contribute to immune dysregulation during COVID-19, possibly favoring disease severity, and suggest that ADO may be a therapeutic approach for the disease.

## Introduction

Coronavirus Disease 2019 (COVID-19) is an inflammatory disease caused by the infection with the SARS coronavirus 2 (SARS-CoV-2). Clinical manifestations of the disease can range from no symptoms or mild upper airway symptoms to severe lower airway symptoms that can evolve to acute respiratory distress syndrome and death (1, 2). Despite the preferential tropism to the lungs, SARS-CoV-2 can be detected in several organs, triggering exacerbated inflammatory responses not only in the target tissues but also systemically, which seems to be responsible for multi-organ failure (3, 4).

Adenosine triphosphate (ATP) is a nucleotide that is present in high concentrations in the cytosol and can be released into the extracellular space upon cellular activation or death (5–7). Extracellular ATP (eATP) can act as a “danger” signal, promoting immune cell activation and eliciting proinflammatory responses (6, 8). Alternatively, eATP can be converted into adenosine (ADO) by ectonucleoside triphosphate diphosphohydrolases (ENTPDases), ecto-nucleotide pyrophosphatases/phosphodiesterases (ENPPs), ecto-5'-nucleotidases, and alkaline phosphatases expressed in the membrane of several immune cells (9–11). Among them, the ectonucleotidase CD39 (ENTPD1) dephosphorylates ATP and ADP to AMP, which is subsequently converted to ADO by CD73 (NT5E) (11). While eATP has proinflammatory properties, ADO can promote immunosuppression *via* inhibition of T cell proliferation and function and induction of anti-inflammatory cytokines (12, 13).

Alterations in the expression and frequency of CD39 and CD73 in leukocytes have already been reported during viral infections and seem to contribute to the inflammatory immunopathology of those diseases (14–16). In addition, it has been reported that viral infection and the viral load itself might influence the purinergic signaling in different viral infections (17, 18). In COVID-19 patients, specifically, there is evidence that the expression of ectoenzymes is modified in T cell lymphocytes and monocytes (19–22). However, whether alterations in this pathway might contribute to the exacerbated inflammatory response associated with COVID-19 is still unclear.

Here we suggest that purinergic signaling might contribute to disease severity during SARS-CoV-2 acute infection. We present evidence to support that the decreased expression of ectonucleotidases in COVID-19 patients’ blood compromises the hydrolysis of ATP in ADO and, together with the reduction in ADO receptors, might favor systemic inflammation. Consistently, ADO partially attenuates inflammatory responses in patients’ cells activated *in vitro*, suggesting that ADO can be considered as a potential therapeutic intervention for COVID-19.

## Materials and methods

### Human subjects

A total of 88 patients with COVID-19 hospitalized at Hospital das Clínicas da Faculdade de Medicina da Universidade de São Paulo (HC-FMUSP) from May 2020 to August 2021 were enrolled in this study. The participants were not vaccinated against COVID-19 and diagnosis was confirmed by reverse-transcriptase polymerase chain reaction (RT-PCR). Patients were divided according to WHO criteria into (1) mild cases, in which no oxygen therapy or oxygen by mask/nasal prong was required, and (2) severe cases, which were submitted to non-invasive ventilation or invasive mechanical ventilation support by the time of sample collection (23). Mild and severe COVID-19 patients had underlying medical conditions including hypertension, diabetes, and obesity, consistent with previously published studies (24, 25). **Supplementary Table 1** summarizes clinical, laboratory, and treatment records from patients. We observed a ∼45.5% mortality rate among patients with severe COVID-19 and ∼9% among those with mild forms of the disease (**Supplementary Figure 1**). We also collected samples from 29 age- and gender-matched healthy unvaccinated controls and with no COVID-19 associated symptoms.

### Sample processing

Blood samples were collected into EDTA tubes and centrifuged at 300 x g for 5 min. Plasma was stored at -80°C for subsequent analysis. The remaining cellular fraction was either stored in RNA Later Solution (Sigma-Aldrich) or processed for leukocyte isolation.

### Gene expression by real-time PCR

Relative gene expression levels of the ectonucleotidases ENPP1, ENPP2, ENPP3, ENTPD1 (CD39), ENTPD5, and NT5E (CD73), as well as the adenosine receptors ADORA1R, ADORA2aR, ADORA2bR, and ADORA3R, were obtained by real-time PCR. Whole blood mRNA was obtained using the RiboPure RNA Purification kit (Thermo Fisher Scientific) and reverse transcribed with the iScript kit (Biorad). For real-time PCR reaction, cDNA was incubated with SYBR Green (Applied Biosystem) and the primers for all target genes, using GAPDH as an internal control. Primers sequences are listed in **Supplementary Table 2**. DNA amplification was carried out in a 7500 Real-time PCR system (Applied Biosystems), and data analysis was performed with the 7500 Software v2.0.6 (Applied Biosystems) according to the delta-CT method (26).

### Plasmatic quantification of nucleotides

ATP and ADO levels in plasma were determined by high-performance liquid chromatography (HPLC), as previously described (27). Briefly, plasma samples were denatured with 0.6 M perchloric acid by centrifugation at 4°C, 16,000 x g for 20 min. After, 4 M KOH was used to neutralize the supernatants, and samples were submitted to second centrifugation. The supernatants were collected and stored at -80°C. Purine levels in the plasma were determined using a reverse-phase HPLC (Shimadzu) using a C18 column (Ultra C18, 25 cm, 4.6 mm, 5 μm, Restek). The elution was carried out using a linear gradient from 100% solvent A (60 mM KH2PO4 and 5 mM of tetrabutylammonium chloride, pH 6.0) to 100% solvent B (solvent A + 30% methanol). The amounts of purines were measured by absorption at 254 nm and the retention times of standards were used as parameters for identification and quantification.

### Immunophenotyping

The expression of CD39 and CD73 in leukocytes was determined by flow cytometry. Samples were incubated with Fc block solution and stained with the following antibodies: anti-CD39 APC (BD-Bioscences), anti-CD73 BB515 (BD-Bioscences), anti-CD3 BV605 (BD-Bioscences), anti-CD14 PerCP (BD-Biosciences) anti-CD19 PE (Beckman Coulter), anti-CD4 V450 (BD-Biosciences) and anti-CD8 V500 (BD-Biosciences). LIVE/DEAD dye (Invitrogen) was used to distinguish dead cells. Samples were fixed with 4% paraformaldehyde and erythrocytes were removed using the FACS Lysing solution (BD Biosciences). Fluorescent cell data acquisition was done using the LSR Fortessa equipment (BD Biosciences) and analyzed with FlowJo software (BD Life Sciences). Fluorescence controls (FMO - Fluorescence Minus One) were realized for all the fluorochromes in the panel.

In cases in which the expression of ADO receptors was evaluated, blood samples depleted of erythrocytes were fixed with 4% paraformaldehyde and permeabilized with Triton-X 100. Cells were stained with primary rabbit polyclonal antibodies against A_1_R, A_2A_R, A_2B_R, and A_3_R (1:500) (Abcam) for 2h at room temperature, followed by incubation with secondary antibody Alexa Fluor 488 goat anti-rabbit (1:1000) (Thermo Fisher). Cells were acquired in an Attune cytometer (Life Technologies) and analyzed with the FlowJo software. Granulocytes and Lymphocytes were differentiated based on the forward/side scatter profile.

### Single-cell data collection and nucleotidases expression analysis

Kazmierski et al. (28) recently performed single cells RNA-Seq experiments investigating the global transcriptional profile of PBMCs from HDs exposed *in vitro* to SARS-CoV-2 for 24h (data accessible at NCBI Gene Expression Omnibus [GEO] accession GSE197665 (28)). We retrieved the data sets to characterize the ectonucleotidase mRNA signature. Analysis of the single-cell RNA data was performed using R Studio v3.6 (R Core Team, 2017) and the Seurat v3.1.4 package (29) and differentially expressed genes were analyzed using the 10x Genomics Loupe Browser (v. 5.0.1).

### Measurement of soluble CD73

The concentration of soluble CD73 in plasma was determined by the human CD73 ELISA kit (NT5E) (Abcam) according to the manufacturer’s instructions. The minimum detection limit was 156 pg/ml.

### Isolation of B cell and the extracellular metabolism of ATP

CD19+ B cells were isolated from blood samples by magnetic separation. Briefly, peripheral blood mononuclear cells were obtained from freshly heparinized blood by density gradient centrifugation. The CD19+ B cells were separated by negative selection using the EasySep Direct HLA B Cell Isolation kit (Stemcell Technologies). The purity of the separated cells determined by flow cytometry exceeded 92%.

B cells were resuspended in phenol red-free RPMI 1640 medium (Gibco) plus 10% fetal bovine serum (FBS) (Sigma-Aldrich) at a concentration of 2.5x10^5^ cells/mL, and 100 µl were plated into a 96-well plate (Jet Bio-Fil). After 18h incubation, the cells were transferred to 1.5ml tubes and resuspended in the incubation buffer containing KCl 5 mM, CaCl2 1.5 mM, EDTA 0.1 mM, glucose 10 mM, sucrose 225 mM, Tris HCl 45 mM, and MgCl2 10 mM, pH 8. ATP (500 µM) (Sigma-Aldrich) was added and cells were incubated for 15, 30, and 60 min. at 37°C and 5% CO2. The reaction was stopped on ice, and cells were centrifuged at 600 x g for 5 min at 4°C. Supernatants were collected and incubated with 10% methanol on ice for 30 min, followed by final refrigerated centrifugation at 25000 x g for 30 min. Samples were stored at -80°C until being analyzed by HPLC as already described.

### Isolation of mononuclear cells

For cell culture experiments, mononuclear cells (MNCs) were obtained by diluting 1mL of buffy coat from EDTA tubes into 11mL of PBS. Samples were layered over a buffered 60% solution of Percoll PLUS reagent (GE Healthcare) and centrifuged at 800 × g for 30 min. Cells at the interphase were collected and counted for the next assays.

### PKA activity assay

MNCs were resuspended in supplemented medium at a concentration of 1x10^6^ cells/mL and were plated into a 48-well plate. After 18h incubation, either ATP or ADO (100µM) (Sigma-Aldrich) were added to the cultures for 30min. Total cellular proteins were extracted and 30ng were used to access the protein kinase A (PKA) activity using the PKA Kinase Activity Assay kit (Abcam) according to the manufacturer’s instructions. The absorbance was measured at 450 nm with a microplate reader ELx800 (BioTek).

### ADO immunomodulation assay

The immunomodulatory capacity of ADO was verified *in vitro*. In brief, 2x10^5^ cells/mL MNCs were cultured in RPMI 1640 medium plus 10% human serum-supplemented or not with ADO (100 µM) for 2h. Cells were further activated using the TLR7/TLR8 agonist CL097 (5 µg/mL) (*In vivo*gen), used as a viral mimetic, for additional 22h. After stimulation, the supernatants from the cultures were stored at -80°C.

### Cytokines measurement

Commercial ELISA kits were used to measure IL-6, TNF-α, and IL-10 (R&D Systems) production in culture supernatants, according to the manufacturer’s protocols. The absorbance was measured at 450 nm with a microplate reader ELx800 (BioTek).

### Statistical analysis

Comparisons between patients and healthy controls were performed with the One-way ANOVA test or Mann-Whitney U test, while for comparisons between paired baseline and stimulated conditions within the same group the Wilcoxon signed-rank test was applied. The Spearman test was used for the correlation analysis. The level of significance considered was p≤0.05.

## Results

### Impaired expression of nucleotidases is associated with inflammatory responses in COVID-19

**Figure 1:**
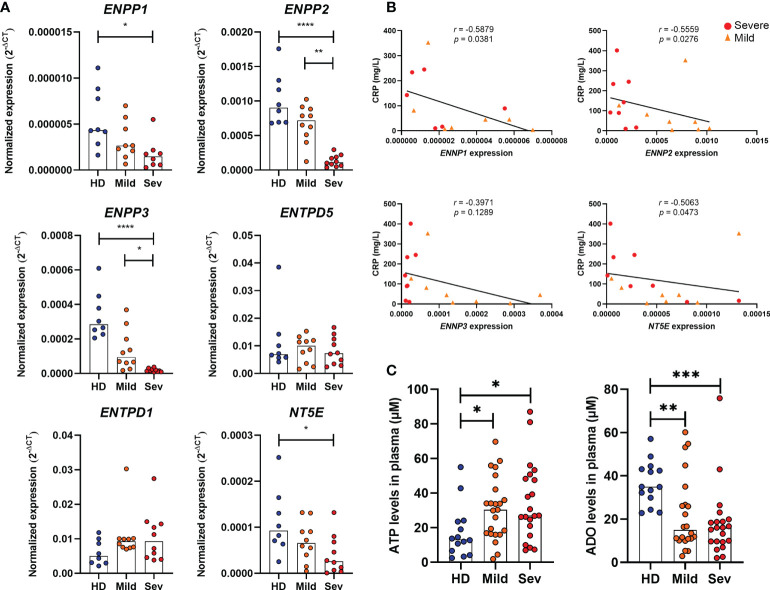
Altered expression of nucleotidases and purinergic composition in the blood of COVID-19 patients. **(A)** Gene expression of nucleotidases ENPP1, ENPP2, ENPP3, ENTPD5, ENTPD1 (CD39) and NT5E (CD73) in whole blood of healthy donors (n=8) and COVID-19 patients (Mild hospitalized, n=10; Severe, n=10). **(B)** Negative correlation between the expression of nucleotidases and blood levels of C-reactive protein (CRP) in COVID-19 patients. **(C)** Plasma levels of ATP and ADO in healthy donors (n=14) and COVID-19 patients (Mild hospitalized, n=23; Severe, n=21). Data are shown as the median. One-way ANOVA test: *p<0.05, **p<0.01, ***p<0.001, ****p<0.0001. Spearman’s correlation test was used to determine the correlation coefficient (r) and the significance (p<0.05). Blue dots indicate healthy donors (HD) whereas orange and red dots indicate hospitalized patients with mild and severe (Sev) COVID-19, respectively.

Although CD39 and CD73 are widely studied in immune cells, several nucleotidases are involved in the extracellular metabolism of purines and hydrolysis of ATP into ADO. Herein, we detected the gene expression of ENPP1, ENPP2, ENPP3, ENTPD1 (CD39), ENTPD5, and NT5E (CD73) in the whole blood of COVID-19 patients. As shown in **Figure 1A**, there is a significant reduction in the expression of *ENPP1*, *ENPP2*, *ENPP3*, and *NT5E* in the blood of patients with severe COVID-19 when compared to HDs and a lower expression of *ENPP2* and *ENPP3* when compared to mild hospitalized cases. Interestingly, the impaired gene expression of these enzymes is negatively correlated to plasmatic levels of C-reactive protein (**Figure 1B**) and the neutrophil-to-lymphocyte ratio (**Supplementary Figure 2**), two inflammatory markers of the disease (30, 31), which is in agreement with the idea that the reduced expression of ectonucleotidases is at least in part accountable for the inflammatory status of COVID-19. In accordance with this data, there is a higher concentration of plasmatic ATP in patients with COVID-19 when compared to HDs, independent of disease severity (**Figure 1C**). At the same time, COVID-19 patients had lower plasma levels of ADO, indicating that the purinergic degradation pathway and ADO production could be compromised in the disease.

### Altered frequencies of CD39+ and CD73+T and B lymphocytes compromise the hydrolysis of extracellular ATP in COVID-19

**Figure 2:**
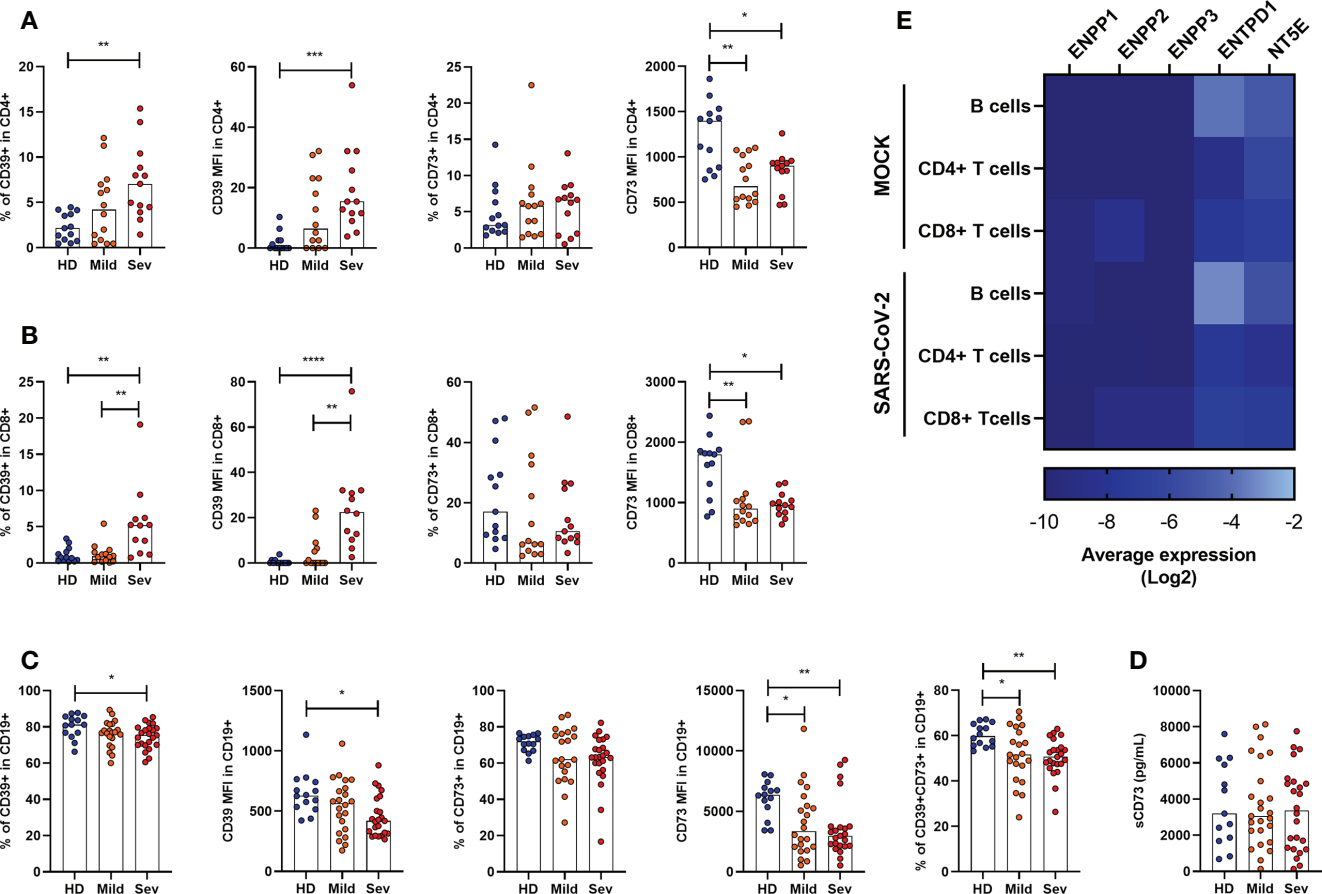
Altered frequency of CD39+ and CD73+ leucocytes in the blood of COVID-19 patients. Frequency and expression of CD39 and CD73 in **(A)** CD4+ T cells, **(B)** CD8+ T cells and **(C)** CD19+ cells from healthy donors (n=14) and COVID-19 patients (Mild hospitalized, n=14-21; Severe, n=12-24) based on the percentage of positive cells and the median of fluorescence (MFI) values. **(D)** Concentration of plasmatic CD73 from healthy donors (n=13) and COVID-19 patients (Mild hospitalized, n=25; Severe, n=24). **(E)** Heatmap of average gene expression values for ENPP1, ENPP2, ENPP3, ENTPD1, and NT5E in B cells, CD4+ T cells, and CD8+ T cells exposed or not to SARS-CoV-2. One-way ANOVA test: *p<0.05, **p<0.01, ***p<0.001, ****p<0.0001. Blue dots indicate healthy donors (HD) whereas orange and red dots indicate hospitalized patients with mild and severe (Sev) COVID-19, respectively.

As the expression of ectonucleotidases varies among leukocytic populations (32–34), we further analyzed the presence of CD39 and CD73 in specific cell types by flow cytometry. It has already been reported that under healthy conditions CD39 is largely expressed on B cells and monocytes, followed by CD4+ T cells and, less significantly, by CD8+ T and NK cells (35). Despite the considerable expression of CD39 in monocytes, we did not observe significant changes in the frequency of CD14+CD39+ cells in SARS-CoV-2 infected individuals (**Supplementary Figure 3**). However, there is an increase in the frequency of CD4+CD39+ and CD8+CD39+ T cells with greater expression of CD39 in patients with severe COVID-19 (**Figures 2A, B**), indicating the prevalence of activated T cells or even regulatory T cells in infected subjects.

On the other hand, CD73 expression is reduced in both, CD4+ and CD8+ T cells, in COVID-19 patients (**Figures 2A, B**), corroborating with previous studies (20). Indeed, while T cell activation leads to upregulation of CD39, it also results in the loss of CD73 from the cell membrane which can remain enzymatically active as a soluble protein. To access if the reduced expression of CD73 in lymphocytes of COVID-19 patients is due to its shedding from the cellular membrane, we quantified soluble CD73 (sCD73) in plasma. As can be seen in **Figure 2D**, there are no differences in the plasma levels of sCD73 between HDs and COVID-19 patients.

While mostly human CD4+ and CD8+ T cells can either express CD39 or CD73, B lymphocytes are usually double-positive for these enzymes and, therefore, play a significant role in the generation of ADO from ATP (36). Interestingly, there is a reduction in the expression of CD39 and CD73 in B cells from COVID-19 patients and the CD19+CD39+CD73+ population is diminished in patients regardless of the disease status (**Figure 2C**).

We further asked if the modified expression of nucleotidases in leukocytes would be a direct effect of viral exposure. Therefore, we reanalyzed single-cell RNA-sequencing data from PBMCs of healthy donors exposed *in vitro* to SARS-CoV-2, as described by Kazmierski et al. (28). Overall, no significant modulation in the expression of *ENPP1*, *ENPP2*, *ENPP3*, *ENTPD1*, and *NT5E* was found in B cells, CD4+ T cells, and CD8+ T cells when they were incubated with SARS-CoV-2 for 24h (**Figure 2E**). Although limited to isolated PBMCs, these data indicate that the altered expression of ectonucleotidases observed in COVID-19 patients is more likely to be induced by the immune response triggered by SARS-CoV-2 than the virus itself.

**Figure 3:**
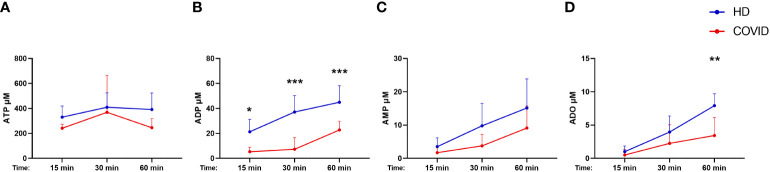
Decreased hydrolysis of ATP by COVID-19 patients’ B cells. Isolated B cells from healthy donors (n=7) and COVID-19 patients (Severe, n=6) were incubated with 500 μM of ATP for 15, 30, and 60 minutes. Consumption of ATP **(A)** and production of ADP **(B)**, AMP **(C)**, and ADO **(D)** was accessed. Data shown as mean with SD. Two-way ANOVA with Bonferroni’s post-test for multiple comparisons: *p<0.05, **p<0.01, ***p<0.001. Blue lines indicate healthy donors (HD) whereas red lines indicate patients with severe (Sev) COVID-19.

Considering that B cells play a crucial role in controlling purinergic-dependent immune responses due to the high expression of CD39 and CD73, the reduction of CD19+CD39+CD73+ cells could directly impact ADO availability. To verify if these phenotypic changes would compromise the consumption of ATP, ADP, and AMP, B cells were isolated from severely ill patients with COVID-19 and HDs and incubated with ATP (500 µM) for various periods. As expected, B cells from subjects infected with SARS-CoV-2 converted less ATP into ADP and produced less ADO when compared with those from HDs (**Figure 3**), indicating an impaired capacity of hydrolyzing ATP in B cells from patients with severe COVID-19.

### ADO signaling is compromised in COVID-19 patients

**Figure 4:**
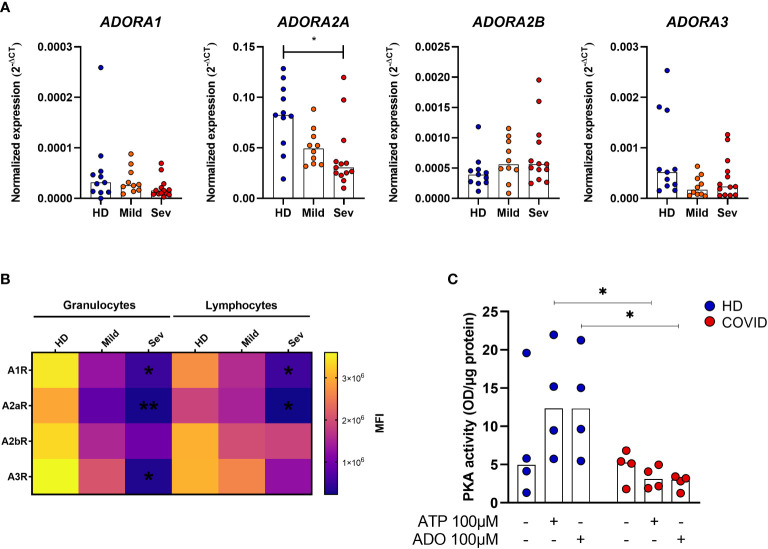
Impaired ADO signaling in COVID-19 patients. **(A)** Gene expression of ADORA1, ADORA2A, ADORA2B, and ADORA3 in whole blood of healthy donors (n=11) and COVID-19 patients (Mild hospitalized, n=10; Severe, n=13). Data presented as median. One-way ANOVA test: *p<0.05. **(B)** Heatmap of A_1_R, A_2A_R, A_2B_R, and A_3_R MFI values expression in lymphocytes and granulocytes of healthy donors (n=6) and COVID-19 patients (Mild hospitalized, n=4; Severe, n=8) based on the median of fluorescence (MFI) values. Data presented as mean. One-way ANOVA test: *p<0.05, **p<0.01. **(C)** MNCs from healthy donors (n=4) and COVID-19 patients (n=4) were incubated with ATP (100 μM) or ADO (100 μM) for 30 minutes. The PKA activity is shown. Data presented as median. Mann-Whitney U test: *p<0.05 (HD vs. COVID-19). Blue dots indicate healthy donors (HD) whereas orange and red dots indicate hospitalized patients with mild and severe (Sev) COVID-19, respectively.

ADO signals *via* receptors A_1_R, A_2A_R, A_2B_R, and A_3_R, and it has been reported that alterations in the expression of these receptors may directly influence the inflammatory response in some pathologies (37, 38). Therefore, we have also investigated the expression of ADO receptors in whole blood samples from COVID-19 patients. Our data indicate a significant reduction of *ADORA2A* gene expression in the whole blood of patients critically ill with COVID-19 (**Figure 4A**). Cell-type flow cytometry analysis additionally suggests that all four ADO receptors are less expressed in both, granulocytes and lymphocytes of COVID-19 patients in a severity-dependent manner (**Figure 4B**).

Upon ADO binding, A_2A_R and A_2B_R couple with the GαS protein and lead to an increase of intracellular cAMP and PKA activity (12, 39). To explore if this signaling pathway is affected during acute COVID-19, we accessed PKA activation in MNCs from patients and HDs. Our findings indicate that MNCs from COVID-19 patients have, indeed, reduced PKA activity rates under ATP or ADO stimulation when compared to HDs cells (**Figure 4C**). Together, these results indicate that not only the generation of ADO from ATP is compromised during COVID-19 but the ADO signaling pathway as well.

### Inhibition of inflammatory responses by ADO

**Figure 5:**
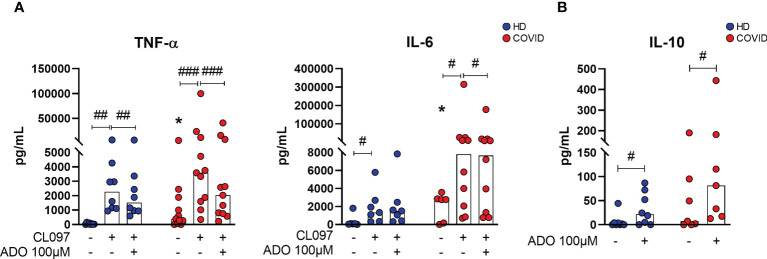
ADO prevents cellular activation triggered by TLRs and induces anti-inflammatory responses. **(A)** MNCs from healthy donors (n=8) and COVID-19 patients (n=11) were incubated with ADO (100 μM) for 2 hours followed by activation with TLR7/8 agonist. Cytokine production in the supernatant after 24h is shown. **(B)** MNCs from healthy donors (n=8) and COVID-19 patients (n=7) were incubated with ADO (100 μM) for 24 hours and IL-10 production in the supernatant after 24h was accessed. Data are shown as the median. Wilcoxon signed-rank test: ^#^p<0.05; ^##^p<0.01, ^###^p<0.001 (between different treatments). Mann-Whitney U test: *p<0.05 (HD vs. COVID-19). Blue dots indicate healthy donors (HD) whereas red dots indicate patients with COVID-19.

In order to investigate whether lower availability of ADO could contribute to the pro-inflammatory profile of COVID-19, MNCs from patients with severe COVID-19 and HDs were cultured with a TLR7/TLR8 agonist (CL097, imidazoquinoline-derived compound) under the presence or absence of ADO. This agonist was chosen as an attempt to mimic innate immune responses triggered during SARS-CoV-2 recognition by MNCs (40). The immunomodulatory effect of ADO was measured by the production of cytokines in the culture supernatants. As shown in **Figure 5A**, incubation with the TLR7/TLR8 agonist induces the production of TNF-α and IL-6 in cells from HDs and COVID-19 patients. Interestingly, in the presence of ADO, this inflammatory response is partially controlled, suggesting a potential anti-inflammatory effect of ADO. Consistent with these findings, we noticed that ADO itself triggers the production of the anti-inflammatory cytokine IL-10 in MNCs from COVID-19 patients and HDs (**Figure 5B**), indicating a possible mechanism by which ADO suppresses the production of inflammatory molecules. Taken together, these data suggest that although the ADO signaling pathway is compromised in COVID-19, this nucleotide can still trigger the production of IL-10 and partially suppress inflammatory responses.

## Discussion

ATP dephosphorylation into ADO mediated by ectonucleotidases is a key regulatory mechanism of immune responses, promoting the shift from ATP-driven inflammation to immunosuppression induced by ADO (41). Given the pronounced inflammatory characteristics of severe COVID-19 and the role of purinergic signaling in immunosuppression, alterations in the metabolism of extracellular nucleotides could contribute to the immunopathogenesis of the disease. Herein, we demonstrate that impaired expression of nucleotidases and lower ADO concentration in the blood is associated with a worse prognosis of COVID-19, while *in vitro* administration of exogenous ADO helps to prevent inflammatory responses in the leukocytes of patients.

Expression profiles of ectonucleotidases can be modified under pathological conditions. Higher expression of CD39 in lymphocytes, for example, has been reported in solid tumors and chronic viral infection by HIV and HCV, and more recently during acute infection by SARS-CoV-2 (21, 42, 43). Induction of CD39 expression occurs upon cellular activation and is regulated by hypoxia, oxidative stress, and inflammatory cytokines such as IL-6 and TNF-α (41, 44–46), which are frequently increased in COVID-19 patients (47–50). Although higher expression of CD39 in T cells from COVID-19 patients might indicate a possible mechanism to counterbalance the inflammatory responses *via* consumption of ATP, its expression in B cells, the major leukocyte population that expresses CD39, is diminished.

The compromised expression of *ENPP1*, *ENPP2*, *ENPP3*, and *NT5E* (CD73) in the peripheral blood of COVID-19 patients shown here, negatively correlates to plasma levels of the inflammatory marker CRP and the neutrophil-to-lymphocyte ratio, supporting the hypothesis of a direct contribution of the purinergic metabolism to the pathogenesis of the disease. In accordance with our findings, Ahmadi et al. reported that loss of CD73 expression in CD8+ T and NKT cells of COVID-19 patients negatively correlates with serum levels of ferritin, another inflammatory marker of the disease (20). It is important to mention, though, that gene expression analysis in whole blood of COVID-19 patients should be interpreted with caution due to the mono-lymphopenia associated with the disease (51, 52). In this context, it is hard to distinguish whether the altered gene expression results from transcriptional events or whether it is a consequence of the imbalance proportion of lymphocytes and monocytes that express such enzymes in the blood of patients. In any case, reduced expression of nucleotidases in peripheral blood may be possibly responsible for the lower concentrations of ADO in the plasma of COVID-19 patients shown here.

Deep and cell-specific analysis evidence that the surface expression of CD73 is impaired in CD4+ T cells and CD8+ T cells in patients with COVID-19, corroborating previously published data (20, 21). In addition, we verified, for the first time, lower expression of CD73 also in CD19+ B cells. Loss of surface CD73 can be explained by alterations at the transcriptional level or also by the shedding of the enzyme from the cell membrane upon cellular activation (53). Complementary, our single-cell RNA-sequencing data analysis from PBMCs exposed to SARS-CoV-2 indicate that the expression of nucleotidases is not likely to be directly affected by the virus (**Figure 2E**). However, it has been shown that incubation with plasma from COVID-19 patients inhibits the expression of CD73 in lymphocytes from HDs (21), suggesting the contribution of soluble factors in these alterations besides the virus itself. Moreover, matrix metalloproteinase (MMP-9), which is released by neutrophils during acute lung damage and is elevated in COVID-19 patients’ blood (54, 55), can cleave CD73 from the cellular membrane generating a soluble protein (56, 57). In our cohort, however, no differences regarding soluble CD73 in the plasma of COVID-19 patients and HDs were observed (**Figure 2D**). Regardless of the specific mechanism behind this alteration has not been elucidated in detail here, loss of CD73 could contribute to the maintenance of the effector function of T cells by preventing ADO-mediated immunosuppression (53, 57). In support of our findings, lymphocyte activation markers such as CD38, CD69, and CD44 are highly expressed on CD4+ and CD8+ T cells of COVID-19 patients (58), and CD73 absence in CD8+ T cells induces granzyme production in these individuals (20).

Different from other lymphocytes, the majority of B cells express CD39 and CD73, contributing significantly to the generation of ADO and inhibiting proliferation and cytokine production in T cells (36). Herein we evidence that B cells from patients with severe COVID-19, which have lower expression of CD39 and CD73, show an impaired capacity to hydrolyze ATP. Similar results were already reported in patients infected with HIV and HBV, where the compromised generation of ADO is claimed to favor inflammatory responses and immune activation (15, 16, 59). Therefore, we have evidences to support the hypothesis that the marked reduction of CD39+CD73+ B cells, together with the impaired expression of CD73 in T cells and the lack of other nucleotidases in the blood, lead to the lower concentrations of plasmatic ADO in COVID-19 patients and might exacerbate innate immune activation. In addition, the absence of CD73 and defective activation of the ADO downstream PKA-mediated phosphorylation of activation-induced deaminase (AID) impairs immunoglobulin class switching in human B cells (60, 61). Whether these alterations compromise humoral responses in COVID-19 patients remains uncertain.

Like us, others have suggested that increased systemic levels of ATP are likely to be involved in the immunopathogenesis of COVID-19 (62, 63), possibly as consequence of the altered expression of nucleotidases. Indeed, the accumulation of ATP has been shown the trigger inflammatory responses such as the activation of the inflammasome pathway (64, 65). Interestingly, there is a higher activation of the NLR family PYRIN domain containing-3, NLRP3, inflammasome in COVID patients (66, 67). In addition, extracellular ATP can contribute to lung local inflammation by recruiting eosinophils, dendritic cells, and neutrophils via P2Y_2_ receptor (68–70), implying that imbalanced metabolization of this nucleotide could directly contribute to the immunopathogenesis of COVID-19.

Apart from the defective hydrolysis of ATP, ADO signaling itself is apparently compromised in COVID-19, where lower expression of ADO receptors and reduced activity of PKA were observed. Mechanistically, activation of the cAMP/PKA pathway *via* ADO receptors inhibits the production of TNF-α, IFN-γ, and IL-2 and T cell proliferation (71, 72). At the same time, ADO receptor signaling, more specific A_2A_R and A_2B_R, has been shown to trigger IL-10 production via CREB activation (73–75). Therefore, the compromised generation of ADO by leukocytes, especially B cells, associated with impaired signaling mediated by ADO receptors could exacerbate inflammatory responses systemically.

Although ADO signaling is compromised in leukocytes of severe COVID-19 patients, we verified that *in vitro* treatment can attenuate the production of TNF-α and IL-6 in CMNs after activation. Both cytokines are increased in the blood of COVID-19 patients (76). In fact, it has been reported that ADO reduces NF-κB activation in T cells and monocytes of COVID-19 patients *in vitro* (21), and the administration of an A2aR agonist attenuated the production of pro-inflammatory cytokines in SARS-CoV-2 mice infection model (77). Moreover, preliminary data suggest that inhaled ADO reduces the levels of CRP in the blood while improving oxygenation rates and reduces hospitalization time in patients with COVID-19 (78, 79). Here we observed that, at the dose used, extracellular ADO can partially overcome the impaired expression of its receptors and imbalanced induction of PKA activity (**Figures 4**, **5**), attenuating inflammatory responses. It is important to mention though that co-treatment with other cAMP inducers, such as the neuropeptide PACAP (80), could result in a more pronounced immunosuppressant effect. Taken together, these findings evidence the potential of ADO as a therapeutic strategy to overcome the exacerbated inflammatory responses in COVID-19.

In summary, our findings indicate that alterations in the purinergic signaling contribute, at least in part, to the immune activation and worse prognosis in acute COVID-19 and reveal the therapeutic potential of ADO-mediated responses in this disease. Whether these alterations persist in recovered patients or impact the development of post-COVID-19 syndrome remains to be elucidated.

## Data availability statement

The authors acknowledge that the data presented in this study must be deposited and made publicly available in an acceptable repository, prior to publication. Frontiers cannot accept a manuscript that does not adhere to our open data policies.

## Ethics statement

The studies involving human participants were reviewed and approved by the Ethics Committee of the HC-FMUSP (no. 30800520.7.0000.0068-2020) and the procedures were conducted following the Declaration of Helsinki. The patients/participants provided their written informed consent to participate in this study.

## Author contributions

AP and MS developed the overall study design, analysis, and manuscript writing. AP, RC, LMO, contributed to data generation and analysis. AP, FT, contributed to the sample processing and data generation. RA, JS, and TG helped to design and execute the chromatography experiments; C. provided the laboratory for patient’s sample manipulation. JK and CG provided single-cell data and performed the analysis; AT, TY, VA, GB recruited and approached patients. FF, AB, and HU helped to design experiments, discussed hypotheses and edited the manuscript. AD provided tools for proper work development. MS directed the study; and all authors reviewed the manuscript.

## Funding

This work was supported by the Laboratory of Dermatology and Immunodeficiencies (LIM-56), the São Paulo Research Foundation (FAPESP) grant numbers 2020/13148-0 and 2018/07366-4, the Coordenação de Aperfeiçoamento de Pessoal de Nível Superior (CAPES) grant number 88887.503842/2020-00 and the National Council for Scientific and Technological Development (CNPq).

## Conflict of interest

The authors declare that the research was conducted in the absence of any commercial or financial relationships that could be construed as a potential conflict of interest.

## Publisher’s note

All claims expressed in this article are solely those of the authors and do not necessarily represent those of their affiliated organizations, or those of the publisher, the editors and the reviewers. Any product that may be evaluated in this article, or claim that may be made by its manufacturer, is not guaranteed or endorsed by the publisher.
