# Supplementary material for: Dysfunctional purinergic signaling correlates with disease severity in COVID-19 patients
Source: Front Immunol. 2022 Sep 30;13:1012027. doi: 10.3389/fimmu.2022.1012027 (PMC9562777; doi:10.3389/fimmu.2022.1012027)
Supplement: Supplementary file 1 [file DataSheet_1.docx]

**Supplementary materials**

**Dysfunctional purinergic signaling correlates with disease severity in COVID-19 patients**

**Supplementary table 1**. Characteristics of COVID-19 patients.

| **Underlying medical condition** | | **Mild** | **Severe** |
| --- | --- | --- | --- |
| Hypertension | | 59.09% | 63.63% |
| Diabetes | | 40.9% | 40.9% |
| Heart disease | | 25% | 25% |
| Obesity | | 22.73% | 20.45% |
| History of smoking | | 20.45% | 22.73% |
| Kidney disease | | 15.9% | 11.36% |
| Thyroid disease | | 13.64% | 9.09% |
| Lung disease | | 9.09% | 11.36% |
| Metabolic disease | | 4.55% | 15.91% |
| Transplant | | 11.36% | 6.82% |
| History of alcohol consumption | | 9.09% | 6.82% |
| Autoimmune disease | | 6.82% | - |
| HIV | | 6.82% | - |
| **Laboratorial findings** | **RV** | **Mild** | **Severe** |
| C-reactive protein (mg/L) | <5.0 | 76.99 | 146.82***** |
| D-Dimer (ng/mL FEU) | <500 | 1799.77 | 9350****** |
| Ratio Neutrophil/Lymphocyte | - | 5.05 | 13.42******** |
| **Medication** | | **Mild** | **Severe** |
| Antibiotics | | 88.64% | 100% |
| Anticoagulant | | 81.82% | 90.91% |
| Corticoid | | 54.55% | 61.36% |
| Antiviral | | 22.73% | 34.09% |
| Antifungal | | 2.27% | 25% |

RV = Reference value.

Data presented as mean. Mann-Whitney U test: *p<0.05, **p<0.01, ****p<0.0001 (Mild vs. Severe).

**Supplementary table 2 -** Oligonucleotides used in real-time PCR experiments.

| **Target** | **Sequence** |
| --- | --- |
| *ADORA1* | F: TGCACTGGCCTGTTCTGTAG |
|  | R: CTGCCTCTCCCACGTACAAT |
| *ADORA2A* | F: CTGGCTGCCCCTACACATC |
|  | R: TCACAACCGAATTGGTGTGGG |
| *ADORA2B* | F: TGCACTGACTTCTACGGCTG |
|  | R: GGTCCCCGTGACCAAACTT |
| *ADORA3* | F: CCTGGGCATCACAATCCACT |
|  | R: ACCCTCTTGTATCTGACGGTA |
| *ENPP1* | F: TGGGTTGAAACCAAGCTGTGCCA  R: CACGGCAGCATCACAGCGACA |
| *ENPP2* | F: AGGAGGAGCTCGTTCCAG  R: TCCCATCCTTCTGCTCTCTT |
| *ENPP3* | F: GCAGGTGGACCAGTCAGTGCC  R: CCGCTGCTTCAGGCCTTCCA |
| *ENTPD1* | F: GGGGAAAGACGAGGAAAGAG  R: CCCACAGCAAGCAAAGCTA |
| *ENTPD5* | F: GCATTTGCCAACACCTTTTT  R: ACAGGGCTCTCTGTGATGCT |
| *GAPDH* | F: GAAGGTGAAGGTCGGAGT  R: GAAGATGGTGATGGGATTTC |
| *NT5E* | F: GGCACTATCTGGTTCACCGT  R: TTTGGCCTCTTTGAGGAGTG |

F= Forward. R= Reverse.

**Supplementary Figures**

**Supplementary Figure 1. Demographic characteristics of the study cohort.** (A) Gender distribution of healthy donors (HD) (n=29) and hospitalized patients with mild (Mild) (n=44) and severe (Sev) (n=44) COVID-19. (B) Disease outcome among COVID-19 patients. (C) Age distribution considering female and male donors among the different groups. Data are shown as the median. One-way ANOVA test: *p<0.05.


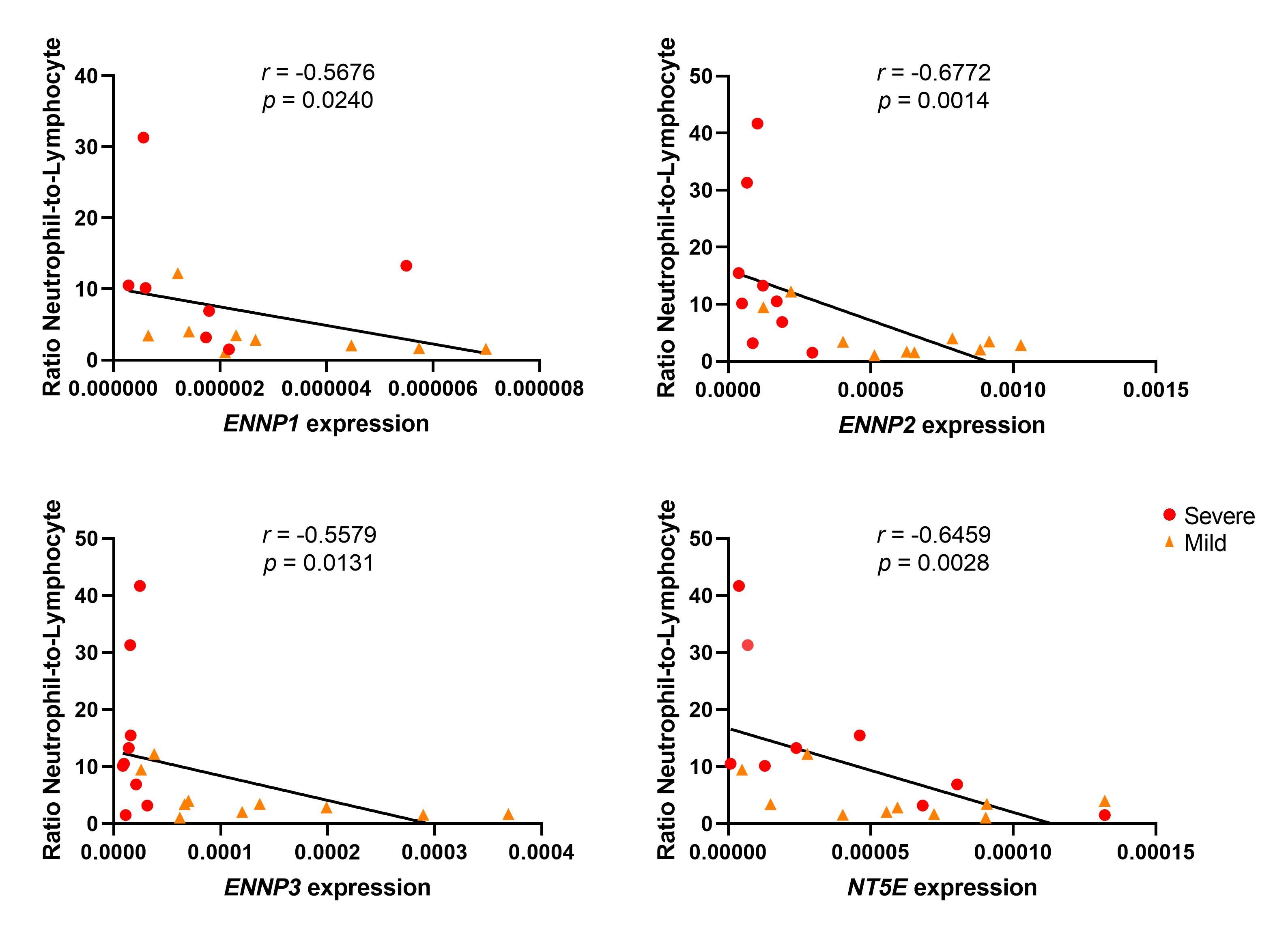


**Supplementary Figure 2. Expression of nucleotidases correlates with** **the neutrophil-to-lymphocyte ratio in COVID-19 patients’ blood.** Negative correlation between the expression of nucleotidases and neutrophil-to-lymphocyte ratio in COVID-19 hospitalized patients (Mild, n=10; Severe, n=9). Spearman’s correlation test was used to determine the correlation coefficient (r) and the significance (p<0.05). Orange triangles indicate patients with mild disease whereas red dots indicate patients with severe COVID-19.


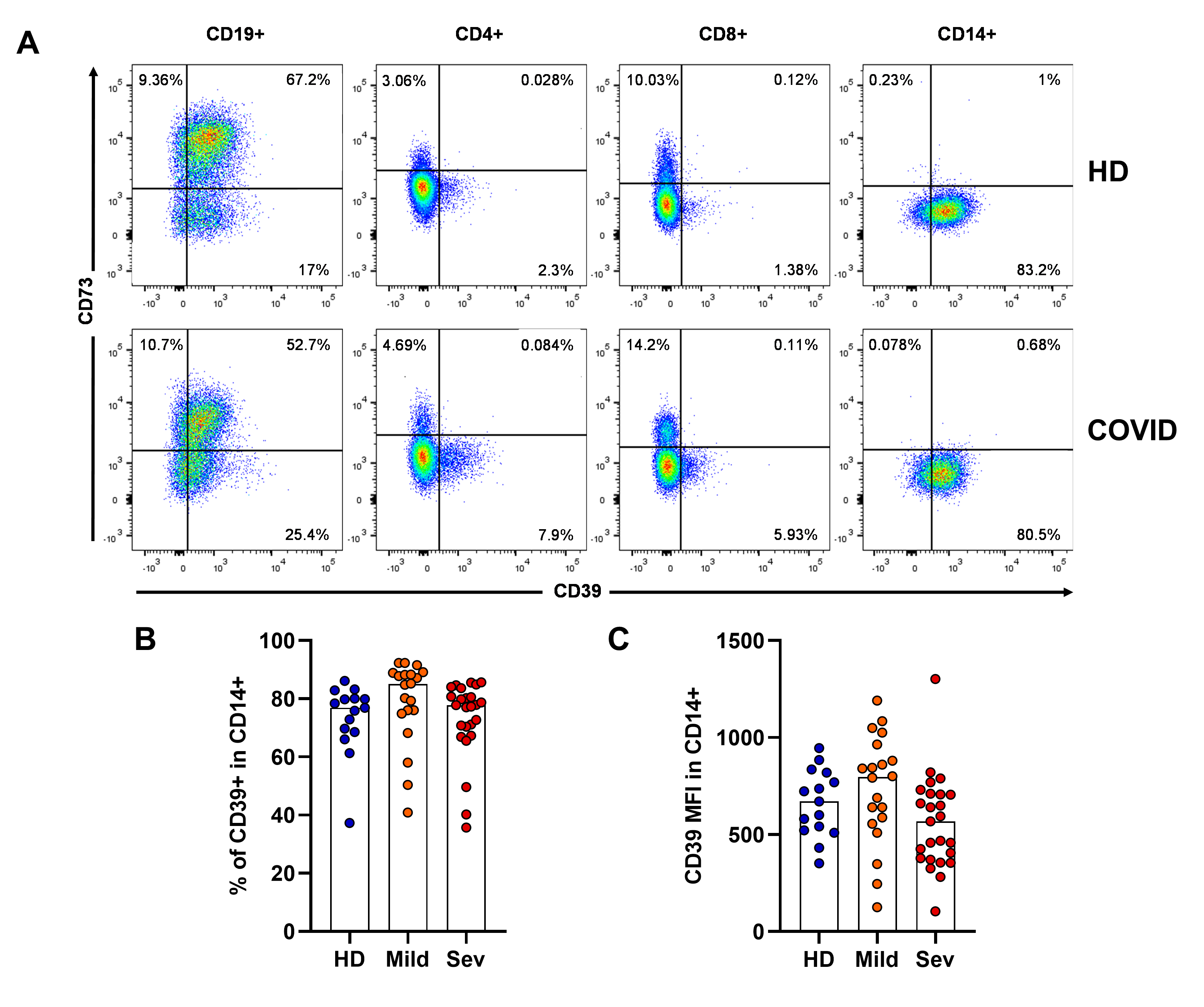


**Supplementary Figure 3. CD39 and CD73 expression among leukocytes.** (A) Representative dot plot indicating CD39 and CD73 gating strategy in different leukocytic populations in healthy donors and COVID-19 patients. (B) Frequency of CD14+CD39+ cells in HD (n=15) and hospitalized patients with mild (n=20) and severe (n=25) COVID-19. (C) Expression of CD39 in CD14+ cells based on the median of fluorescence (MFI) values. Blue dots indicate healthy donors (HD) whereas orange and red dots indicate patients with mild and severe (Sev) COVID-19, respectively.
